# Therapeutic Drug Monitoring Characteristics in a Tertiary University Hospital in 2019

**DOI:** 10.7759/cureus.7612

**Published:** 2020-04-10

**Authors:** Zeynep Günes Ozunal, Belkiz Ongen İpek

**Affiliations:** 1 Medical Pharmacology, Maltepe University Faculty of Medicine, İstanbul, TUR; 2 Medical Biochemistry, Maltepe University Faculty of Medicine, İstanbul, TUR

**Keywords:** biochemistry, carbamazepine, digoxin, lithium, pharmacology, therapeutic drug monitoring, valproic acid

## Abstract

Introduction

Therapeutic drug monitoring (TDM) is defined as measuring drug concentration in a biological sample to optimize pharmacotherapy. This study aims to evaluate TDM requests in a tertiary university hospital retrospectively.

Materials and methods

TDM requests were evaluated retrospectively for lithium, valproic acid, carbamazepine, and digoxin in 2019. The age and gender of the patient, requesting department, and measurement results were evaluated. Lower levels than the reference values were considered as subtherapeutic, while levels higher than the reference were considered as toxic.

Results

A total of 415 drug level measurement records were found. The pediatric age sample ratio was 13.7%, and the elderly age sample ratio was 11.8%. When all samples were evaluated according to the relevant laboratory cut-off values, 72.8% of samples were within the therapeutic level range, 21.9% of samples were subtherapeutic, and 5.3% were toxic. The pediatric age group had a higher ratio of toxic levels for the four drugs studied (54.5%).

Conclusions

Tests for lithium, valproic acid, carbamazepine, and digoxin would not be considered sufficient for TDM. Multidisciplinary teamwork might be appropriate for further implementation and interpretation of TDM.

## Introduction

Therapeutic drug monitoring (TDM) is the clinical practice of measuring the drug concentration in biological samples to optimize pharmacotherapy. It has been used since the 1970s to provide better drug response and avoid adverse effects. Identification of non-compliant patient, personalization of dosage, and investigation of non-response are some benefits of TDM. There are many prescribed drugs, but few have a clinical impact on TDM [1]. Drugs that are candidates for TDM should satisfy pharmacological properties such as a narrow therapeutic index, significant pharmacokinetic variability, presence of a reasonable relationship between drug concentration and clinical effect, and an established target for concentration range [2]. There are forthcoming drugs, for which TDM is accepted as a standard of medical care. Examples are aminoglycoside antibiotics, cardiac glycosides, antiepileptic drugs, digoxin (DIGOX), theophylline, cyclosporin, tacrolimus, methotrexate, and salicylates, when used in high doses [2,3]. TDM requiring drugs might be indicated in epilepsy, mood disorders, heart failure, respiratory disease, and neoplastic or rheumatologic disease treatment. This study aims to evaluate TDM requests retrospectively in a tertiary university hospital.

## Materials and methods

Ethical approval was obtained from the ethical committee of Maltepe University Medical Faculty (issue number: 2019/900/08). TDM requests were evaluated retrospectively from electronic records in 2019. Both inpatient and outpatient data were included in the study. Patient age and gender were obtained from the records. The age data were stratified as 0 to 18 years as pediatric, 18 to 65 years as an adult, and > 65 years as elderly. The requesting department and measurement results were evaluated. Therapeutic ranges were assigned based on routine laboratory ranges. Lower levels than reference values were considered as subtherapeutic, and higher levels than reference values were considered toxic.

All data were expressed as mean ± standard error of the means (SEM) [4]. The evaluation was performed by descriptive statistics and, to assess categorical data, a Pearson's chi-squared test was used. GraphPad Prism V.8.01 (San Diego, CA) and IBM SPSS Statistics for Windows, Version 25.0. (IBM Corp., Armonk, NY) were used for statistical analysis and graphing.

## Results

**Figure 1:**
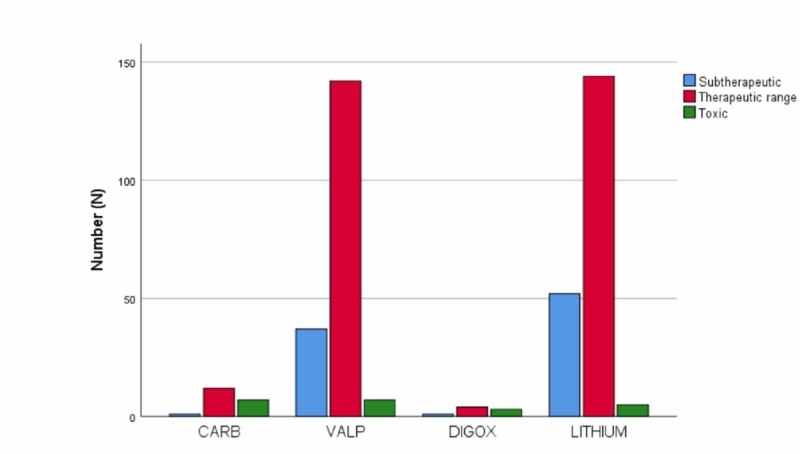
Sample number (N) distribution of therapeutic drug monitoring results to the subtherapeutic, therapeutic range, and toxic level CARB, carbamazepine; DIGOX, digoxin; LITHIUM, lithium; VALP, valproic acid.

A total of 415 drug level measurement records were found for 2019. The mean age was 40.4 years. The pediatric age sample ratio was 13.7%, and the elderly age sample ratio was 11.8%. Fully 74.5% of blood samples were drawn from patients in the adult age group. The gender ratio was 33% men to 67% women. The drugs monitored were lithium (LITHIUM), valproic acid (VALP), carbamazepine (CARB), and DIGOX. When all samples were evaluated according to the relevant laboratory cut-off, 72.8% of the samples were within the therapeutic level range, 21.9% of the samples were subtherapeutic, and 5.3% of the samples were toxic. The most frequent TDM request was LITHIUM, while the least frequent was DIGOX (Table 1). The samples for LITHIUM and VALP TDM resulted 25.9% (52) and 19.9% (37) in the subtherapeutic levels, respectively. The highest number of samples was 144 and in LITHIUM therapeutic range group (Figure 1). DIGOX was within the therapeutic range the least (50%). When age groups (pediatrics, adult, elderly) and drug level groups (subtherapeutic, therapeutic, toxic) were evaluated, the differences were found to be statistically significant. The pediatric age group had a higher ratio of toxic drug levels than the other age groups.

**Table 1:** Therapeutic drug monitoring details by age, gender, plasma drug level, reference range, and distribution of categorization based on the reference range *Reference range of carbamazepine is 4-8 µg/mL in case of concomitant antiepileptic use. SEM, standard error of the mean.

Drug (N)	Age min-max (mean±SEM)	Gender female ratio (%)	Level (mean±SEM) (reference range)	Subtherapeutic level N (%)	Therapeutic range N (%)	Toxic level N (%)
Lithium (201)	10-78 (43.96±1.069)	147/201 (73.1%)	0.73±0.17 (0.6-1.2 mmol/L)	52 (25.9%)	144 (71.6%)	5 (2.5%)
Valproic acid (186)	1-85 (35.66±1.512)	119/186 (64%)	64.23±1.73 (50-100 µg/mL )	37 (19.9%)	142 (76.3%)	7 (3.8%)
Carbamazepine (20)	7-87 (32.45±5.6)	9/20 (45%)	9.06±0,75 (5-12 µg/mL)*	1 (5%)	12 (60%)	7 (35%)
Digoxin (8)	76-91 (83.38±1.90)	4/8 (50%)	2.85±1.27 (0.8-2 ng/mL)	1 (12.5%)	4 (50%)	3 (37.5%)

**Figure 2:**
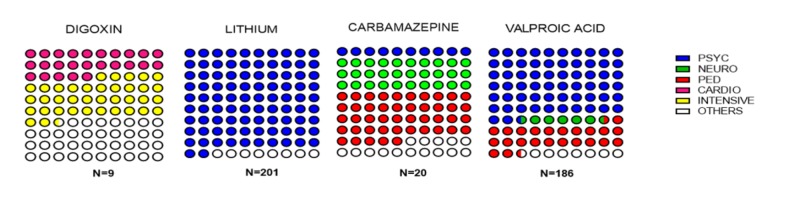
Distribution of departments requesting for therapeutic drug monitoring N refers to the number of samples. CARDIO, cardiology; INTENSIVE, intensive care; NEURO, neurology; PED, pediatrics; PSYC, psychiatry.

The department requesting TDM differed based on the drug (Figure 2). The three departments that most frequently requested drug level measurement were psychiatry (n=301), pediatrics (n=53), and neurology (n=20). Pediatric neurology requests were counted in pediatrics (n=35) and made up 66% of pediatrics requests. On the other hand, VALP and LITHIUM were most frequently requested TDMs by psychiatry specialists, CARB was from pediatrics, and DIGOX was from intensive care (Figure 2). DIGOX level measurements were requested by intensive care specialists (37.5%) and by cardiology specialists (25%).

## Discussion

The present retrospective study involved a one-year evaluation of 415 blood samples for TDM in approximately 200 beds and 15,000 outpatient/years in a tertiary university hospital setting. The drug requests were CARB, VALP, DIGOX, and LITHIUM. The most commonly monitored drugs mentioned were CARP, VALP, and DIGOX; these were among our monitored drugs [2]. The sample size was comparable with a four-year study from Turkey that evaluated a total of 7,759 measurements in a university hospital with more than 2,500 beds [5]. The study results indicated that the pediatric age group had a lower frequency of samples within the therapeutic range. This might be due to the fact that many of the drugs being monitored in the pediatric population were developed based on adult drug pharmacokinetics and disease states, which might be quite different from the pediatric population [6].

VALP was a frequently prescribed drug for TDM in neuropsychiatric disorders, with requests from neurology, pediatrics, and psychiatry departments. Age consideration was important for VALP as its use was not recommended in geriatric patients; however, 8.6% (n=16) of the patients in our study were elderly. The clinical need for VALP in this population should be evaluated as it is potentially inappropriate for older adults. In all age groups, subtherapeutic level results were found in 19.9% of samples. This might be interpreted as undertreatment. VALP metabolism was also altered with CYP2C9 and CYP2A6 polymorphisms [7]. Pharmacogenetics testing might contribute to a better understanding of the pharmacokinetic variability observed between individuals.

LITHIUM was frequently found in our TDM samples. Nearly one-quarter (25.9%) of the results were for subtherapeutic levels, while 71.6% were within the therapeutic range. Even though it was frequent in our study, it was much more than expected. In Japan, regulatory warnings for LITHIUM have been shown to be effective in increasing TDM for the drug [8].

DIGOX, a cardiac glycoside, had the lowest ratio for being in the therapeutic range. Intensive care (37.5%) constituted the primary requesting department. This might indicate that DIGOX was monitored primarily for toxicity in intensive care. In this hospital setting, the results for DIGOX were returned in a few days and might be the reason it did not apply in outpatient settings. Even though TDM for DIGOX was highly recommended in the outpatient setting, a lower than the maximum therapeutic dose may have been believed to be safer for the treatment of chronic heart failure [9].

As an antiepileptic, CARB had the highest ratio of toxic levels and the least number of samples. There was a single CARB result for a sample below the lower limit; that particular sample might have been a request to monitor for adherence to therapy or a differential diagnosis for toxic clinical presentation. Our therapeutic range was only different for CARB in cases of concomitant antiepileptic use. This fact might support that CARB should be better disseminated for monitoring with TDM.

All biological samples were from blood, though other biological fluids might be eligible for drug monitoring. The pediatric age group samples correlated with a lesser degree of being in the therapeutic range. For a better drug response, other biological fluids that can be obtained non-invasively, such as saliva, should be considered for TDM. Saliva samples may provide accurate levels for some drugs and might be chosen in the pediatric age group [10].

Limitations of the study include the lack of information for appropriateness of collecting time, the purpose for requesting TDM, and the presence of concomitant drugs. The time of blood collection, perhaps more than any other factor, can contribute to the misinterpretation of drug levels [11]. In the study, TDM was requested by the relevant departments. The blood collection time accuracy was considered appropriate as patients are informed about the accurate time. There are many indications for TDM, such as monitoring drug use compliance for individualizing pharmacotherapy, diagnosing undertreatment, or checking for drug toxicity [12]. Protein binding was another major factor that varies by drug, but that also can be altered by drug-drug and drug-disease interactions. [11]. Some of the patients were using more than one antiepileptic or other drugs. Time intervals after a drug were taken, drug-drug, drug-disease, and drug-nutrient interactions also should be evaluated for accurate TDM interpretation.

TDM is a valuable tool to ameliorate pharmacotherapy to increase the efficacy and decrease the adverse drug reactions. Most patients had a therapeutic response within the reference range, but many patients might need target concentrations outside the reference range [13]. Interpretation of drug concentrations should involve clinical monitoring for efficacy and adverse drug reactions, and consider drug-drug and drug-disease interactions, as well as variability in pharmacogenetics.

The present study in the university hospital setting prompted psychiatry, pediatrics, neurology, cardiology, and intensive care departments that were more relevant to TDM practice. In these departments, four drugs were requested for TDM. Interestingly, phenytoin, an antiepileptic drug, was not requested even though it was in the recommended drug list for TDM and has clinically important drug-drug interactions with newer antiepileptics [2,13]. TDM of aminoglycoside antibiotics, theophylline, cyclosporin, tacrolimus, methotrexate, and salicylate was not in the requested samples. The TDM reporting duration advised and indicated to be important was preferably within 24 hours of dosing, especially during dosage adjustments, and in diagnosing toxicity [12]. Our laboratory fulfills these criteria only for VALP and LITHIUM; these were found to be the most requested. Our study result yielded that the pediatric age group might be more vulnerable in maintaining a therapeutic range. Non-invasive monitoring with saliva samples for eligible drugs might be an option to maximize TDM.

## Conclusions

In our study, a total of 415 drug level measurement records were found. The drugs monitored were LITHIUM, VALP, CARB, and DIGOX. When the samples were interpreted according to laboratory cut-off, 72.8% of the samples were within the therapeutic level range, 21.9% of the samples were subtherapeutic, and 5.3% of the samples were toxic. A multidisciplinary team approach is essential for appropriate TDM. LITHIUM, VALP, CARB, and DIGOX, as the requested tests in our hospital, would not be considered sufficient for TDM management. Pharmacologic aspects, laboratory analysis, and clinical monitoring are the three main components of the teamwork required. Biochemistry, pharmacology, and relevant departments should connect to interpret TDM for optimizing pharmacotherapy.
